# Prediction of 90-day mortality in older patients after discharge from an emergency department: a retrospective follow-up study

**DOI:** 10.1186/s12873-016-0090-5

**Published:** 2016-07-13

**Authors:** Susanna E. Hofman, Jacinta A. Lucke, Noor Heim, Jelle de Gelder, Anne J. Fogteloo, Christian Heringhaus, Bas de Groot, Anton J. M. de Craen, Gerard Jan Blauw, Simon P. Mooijaart

**Affiliations:** Department of Gerontology and Geriatrics, Leiden University Medical Center, PO Box 9600, Leiden, 2300 RC The Netherlands; Department of Emergency Medicine, Leiden University Medical Center, PO Box 9600, Leiden, 2300 RC The Netherlands; Department of Internal Medicine, Leiden University Medical Center, PO Box 9600, Leiden, 2300 RC The Netherlands; Institute for Evidence-based Medicine in Old Age|IEMO, PO Box 9600, Leiden, 2300 RC The Netherlands

**Keywords:** Emergency medicine, Geriatrics, Risk factors, Mortality

## Abstract

**Background:**

Older people frequently attend the emergency department (ED) and have a high risk of poor outcome as compared to their younger counterparts. Our aim was to study routinely collected clinical parameters as predictors of 90-day mortality in older patients attending our ED.

**Methods:**

We conducted a retrospective follow-up study at the Leiden University Medical Center (The Netherlands) among patients aged 70 years or older attending the ED in 2012. Predictors were age, gender, time and way of arrival, presenting complaint, consulting medical specialty, vital signs, pain score and laboratory testing. Cox regression analyses were performed to analyse the association between these predictors and 90-day mortality.

**Results:**

Three thousand two hundred one unique patients were eligible for inclusion. Ninety-day mortality was 10.5 % for the total group. Independent predictors of mortality were age (hazard ratio [HR] 1.06, 95 % confidence interval [95 % CI] 1.04-1.08), referral from another hospital (HR 2.74, 95 % CI 1.22-6.11), allocation to a non-surgical specialty (HR: 1.55, 95 % CI 1.13-2.14), increased respiration rate (HR up to 2.21, 95 % CI 1.25-3.92), low oxygen saturation (HR up to 1.96, 95 % CI 1.19-3.23), hypothermia (HR 2.27, 95 % CI 1.28-4.01), fever (HR 0.43, 95 % CI 0.24-0.75), high pain score (HR 1.55, 95 % CI 1.03-2.32) and the indication to perform laboratory testing (HR 3.44, 95 % CI 2.13-5.56).

**Conclusions:**

Routinely collected parameters at the ED can predict 90-day mortality in older patients presenting to the ED. This study forms the first step towards creating a new and simple screening tool to predict and improve health outcome in acutely presenting older patients.

## Background

Older patients frequently attend emergency departments (EDs) in comparison with younger adults [1, 2]. Admittance to the ED is associated with risk of negative health outcomes such as functional decline [3] and mortality [2]. However, little is known about predictors of mortality in the period after presentation to the ED in older patients.

Predictors of poor outcome in older patients can be divided into two categories. On one hand, there is the level of vulnerability of the older patient, which is reflected in for instance multi-morbidity, poly-pharmacy, functional capacity and cognitive and social functioning [4]. Frequently studied prediction tools such as the Identification of Seniors At Risk [5] and the Triage Risk Screening Tool [6] are based on these parameters. On the other hand, parameters reflecting severity of disease at presentation may also determine poor outcome [7]. Specific diagnoses are well known predictors of mortality but are very numerous and hard to categorise, partly due to the large heterogeneity of older patients, especially in the presence of multi-morbidity [8]. Other, more generic data on severity of disease are routinely recorded as part of medical practice, e.g., time of arrival [9], vital signs [7] and laboratory parameters [10], and may also predict poor outcome. However, little is known about their association with mortality in older patients in the period after discharge from the ED. Identifying such predictors may enable us to design an adequate screening tool in order to target older patients at high risk of negative health outcome early during ED admittance. A screening tool may enable fast-tracking patients that are likely to be admitted to an inpatient ward and shorten their stay at the ED. In case of high risk of mortality, advanced care planning may be initiated at the ED or shortly after admission, or rehabilitation in case of high risk of functional decline.

Our aim was to study whether routinely recorded parameters in the ED, such as way and time of arrival, vital signs and laboratory results independently predict 90-day mortality. We performed a retrospective follow-up study among patients aged 70 years or older visiting our ED.

## Methods

### Study design

Our study was conducted at the ED of the Leiden University Medical Center, a tertiary university teaching and level 1 trauma hospital in the Netherlands. Patients aged 70 years and older that had attended the ED between 1 January 2012 and 31 December 2012 were included retrospectively. The Medical Ethics Committee of the Leiden University Medical Center waived the obligation of approval as data were collected in the past as part of routine clinical care.

### Health care in the Netherlands

The Netherlands is a small and highly populated country in Europe measuring 41.5 thousand square kilometres [11] and counting 16.7 million people in 2012 [12]. Standard medical care is equally accessible for every Dutch citizen through legally mandatory health insurance. Primary care is provided by general practitioners (GPs). Specialist care can only be accessed after referral by a GP. One of the exceptions are EDs of hospitals, where a substantial proportion of patients are self-referred [13]. The Leiden University Medical Center is a tertiary referral centre in Leiden. The ED is one of two level 1 trauma EDs that together serve a catchment area of 400.000 inhabitants, both urban and rural. The population is predominantly Caucasian and includes all social classes. Our ED is equipped with 15 rooms of which three are specially designed to accommodate trauma victims. Patients are triaged by an ED nurse. Within hours self-referred patients are evaluated by an ED physician or ED resident. Out of hours self-referred patients are primarily evaluated by a GP and if indicated subsequently referred to an ED physician or ED resident. Referred patients are directly allocated to a resident of the appropriate medical specialty present at the ED. After evaluation patients are either treated at the ED and discharged home or admitted to an inpatient ward. Patients with an electrocardiogram indicative for myocardial infarction bypass our ED and are immediately referred to the catheterisation laboratory [14]. As a consequence, they are not included in the present study.

### Selection of study population

Patients were identified in our computerised patient record system (ChipSoft-EZIS®, version 5.2, 2006–2014, Amsterdam, The Netherlands, www.chipsoft.nl). Several steps of exclusion criteria were applied. Our study was aimed at a selection of older patients that may benefit from additional interventions during or following an ED visit. First, medical records based upon unjustified ED use were excluded. Unjustified ED use was defined as ED use for any other reason than acute medical care, such as outpatient check-ups on weekends, plaster cast readjustments, performed blood tests for other medical departments and patients who decided to leave the ED before medical attention was bestowed. We believe these are not representative for the acutely presenting older patient visiting the ED and may disturb associations between predictors and outcome results. Second, patients who deceased in the ED and patients receiving cardiopulmonary resuscitation therapy upon arrival were excluded from analysis since prognosis of these patients is known to be poor and these patients fall outside the scope for identifying new predictors [15]. As we used retrospective data, we were unable to assess whether an ED visit was the first or one of many visits. Patients may have visited other hospitals as well as ours or made visits outside our selected timeframe. Therefore, we included only the first ED visit of each patient in 2012.

### Potential predictors

Apart from demographic characteristics (age and gender), we selected routinely collected parameters that may reflect severity of disease as presented in the acute situation. We investigated time and way of arrival, presenting complaint, consulting medical specialty, vital signs, pain score and laboratory parameters. These data were automatically generated from the digital patient records and outliers were manually checked for validity by a researcher. Triage category was not included since we were interested in universal predictors and hospitals differ in the triage systems they use.

Time of ED visit was determined from ED registration time and subdivided in three categories, day (08.00 h–15.59 h), evening (16.00 h–23.59 h) and night time (00.00 h–07.59 h). Way of arrival at the ED was mutually exclusively noted as self-referral, brought in by ambulance, referral by a GP, internal referral from another department or referral by another hospital. Patients categorised as self-referral or referral by a GP visited the ED with private transportation. By contrast, patients who arrived by ambulance were categorised as brought in by ambulance regardless of whether the ambulance was ordered by a referring GP or because of an emergency call. Dutch ambulance staff is trained to judge the accuracy of emergency calls at the scene. Ambulance staff will only transport such patients to the hospital if they consider the referral justified. At our hospital, triage is based on the Manchester Triage System (MTS) [16]. This system uses flow charts for 55 disease presentations to determine the level of urgency and associated target time a patient should receive care from a physician. The presenting complaints of our study population were categorised according to these MTS disease presentations [16]. Disease presentations occurring in less than 3 % of patients were merged as ‘other’. The medical specialty a patient was assigned to was categorised as surgical or non-surgical [17]. Finally, we listed clinical measurements that were recorded in the ED: vital signs, pain score and laboratory results. At triage, an ED nurse determined which clinical measurements were medically indicated according to protocols. They were measured at triage or soon after a patient was placed into a treatment room. Laboratory testing is performed on indication and either ordered by an ED nurse or consulting physician. The first set of vital signs assessed in the ED was recorded. Vital signs were categorised according to the Modified Early Warning Score and included systolic blood pressure, heart rate, respiratory rate and body temperature [18]. Oxygen saturation was recorded as well [19]. Categories containing less than 1 % of patients were combined with adjacent categories, but not with the reference category, in order to minimise the number of categories. Pain was evaluated using the Numeric Rating Scale (NRS) rating from 0 to 10 and categorised as no or light (NRS 0–3), mild (NRS 4–6) and serious (7–10) pain according to the Dutch guidelines for pain classification in emergency settings [20]. Blood pressure, heart rate, respiratory rate and oxygen saturation were measured using a medical monitor (IntelliVue MP50®, Eindhoven, The Netherlands, www.philips.nl/healthcare). Body temperature was determined by a tympanic thermometer (Genius 2®, Mansfield, USA, www.covidien.com). Registered laboratory results were haemoglobin, thrombocytes, leukocytes, C-reactive protein, sodium, potassium, creatinine, urea, troponin T and non-fasted glucose. Vital signs and laboratory parameters will only be assessed if there is a medical indication to do so. If data on vital signs were missing, they were either not measured or they were measured but not recorded in the medical chart correctly. It is impossible to categorise this in a retrospective manner. Therefore, we assumed that missing vital signs meant that there was no indication to perform these measurements.

### Primary end point

Our primary outcome measure was mortality in the first three months after ED admittance. Beyond this time period, the association of predictors measured at baseline and mortality is likely to be obscured by the occurrence of new medical events. Mortality data were acquired from the municipal personal records database on 1 May 2014.

### Statistical methods

Data are displayed as mean and standard deviation if normally distributed and median and interquartile range if not normally distributed. To investigate the association between predictors and mortality we used Cox proportional hazards models. We performed uni- and multivariate Cox regression analysis. In the univariate models only one parameter was entered as independent variable. In the multivariate analyses, multiple parameters were entered as independent variables simultaneously to assess which were independent predictors of mortality. Our study was aimed at potential predictors assessed upon or soon after arrival at the ED. Results of laboratory testing became available at least one hour after withdrawal, but laboratory testing is usually ordered in the first few minutes after a patient is placed into a treatment room. Therefore, we added merely the medical indication to perform laboratory testing to the set of predictors in the multivariate model. As an in-depth analysis we have additionally analysed the univariate association of individual laboratory results with mortality using univariate Cox regression. The level of significance was set at *P* < 0.05. All statistical analyses were performed using IBM SPSS Statistics package (version 20).

## Results

**Fig. 1:**
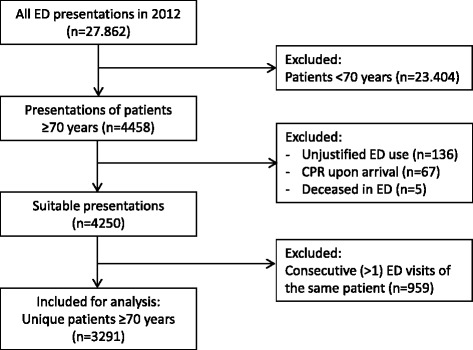
Flow chart of participant selection. Abbreviations. ED = emergency department, CPR = cardiopulmonary resuscitation

During 2012, there were 27.862 Emergency Department (ED) visits of which 4458 (16 %) visits were by patients aged 70 years or older. Visits were excluded because of inappropriate ED use (*n* = 136), receiving cardiopulmonary resuscitation upon arrival (*n* = 67) and patients who deceased in the ED (*n* = 5). This left 4250 suitable ED presentations of which 959 were repeat visits, leaving 3291 unique patients eligible for the analyses (Fig. 1).

**Fig. 2:**
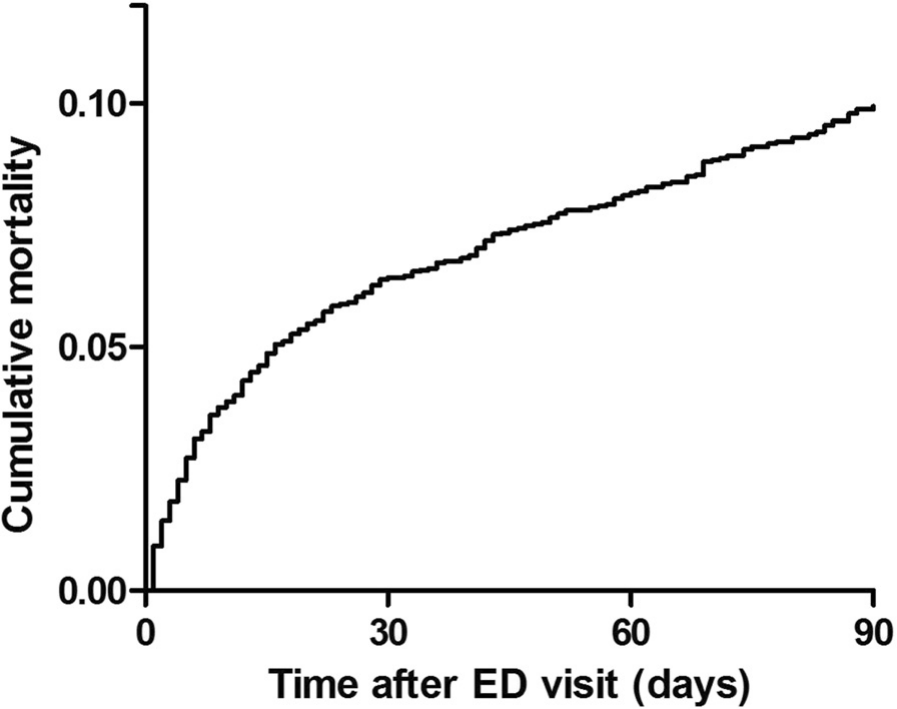
Cumulative mortality in older patients after an ED visit. Abbreviations. ED = emergency department

Baseline characteristics of the study population are described in Table 1. Median age was 78.3 years (interquartile range 74.0–83.6 years) and 53.1 % was female. Most patients arrived by ambulance (35.2 %) or with private transportation after referral by their GP (33.7 %). Patients were assigned to a non-surgical specialty in 58.3 % of cases. Mortality rate at 30 days after ED presentation was 7.0 % and increased to 10.5 % at 90 days after an ED visit (Fig. 2).

**Table 1:** Baseline characteristics of study population

ED Characteristics	All unique patients^a^ (*N* = 3291)
Demographics	
Age, *median (IQR)*	78.3 (74.0–83.6)
Female*, N (%)*	1748 (53.1)
Time of ED visit, *N (%)*	
Day 08.00 h-15.59 h	1677 (51.0)
Evening 16.00 h-23.59 h	1254 (38.1)
Night 00.00 h-07.59 h	360 (10.9)
Way of arrival, *N (%)*	
Self-referral	654 (19.9)
Brought in by ambulance	1159 (35.2)
General practitioner	1108 (33.7)
LUMC internal	338 (10.3)
Other hospital	28 (0.9)
Unknown	4 (0.1)
Presentation, *N (%)*	
Limb problems	608 (18.5)
Unwell	598 (18.2)
Chest pain	346 (10.5)
Shortness of breath	304 (9.2)
Abdominal pain	214 (6.5)
Collapsed	168 (5.1)
Falls	122 (3.7)
Wounds	108 (3.3)
Palpitations	101 (3.1)
Other	722 (21.9)
Consulting medical specialty, *N (%)*	
Surgical	1371 (41.7)
Non-surgical	1920 (58.3)
Vital signs^b^	
Systolic BP (mmHg), *mean (sd)*	146.5 (28.3)
Heart rate (beats/min), *mean (sd)*	83.7 (21.0)
Oxygen saturation (%), *median (IQR)*	98 (3)
Respiration rate (breaths/min), *mean* (*sd)*	18.7 (5.5)
Temperature (°C), *mean (sd)*	36.9 (1.0)
Pain score (NRS)^b^, *median (IQR)*	3 (1–5)
Laboratory results ^b,c^	
Haemoglobin (mmol/L), *mean (sd)*	8.1 (1.2)
Thrombocytes (*10^9^/L), *mean (sd)*	229 (94)
Leukocytes (*10^9^/L), *median (IQR)*	8.75 (6.80–11.41)
C-reactive protein (mg/L), *median (IQR)*	6.0 (0.0–30.0)
Sodium (mmol/L), *mean (sd)*	139 (4)
Potassium (mmol/L), *mean (sd)*	4.3 (0.6)
Creatinine (μmol/L), *median (IQR)*	84 (67–109)
Urea (mmol/L), *median (IQR)*	7.6 (5.9–10.2)
Troponin T (μg/L), *median (IQR)*	0.014 (0.007–0.028)
Non fasted glucose (mmol/L), *mean (sd)*	7.9 (3.3)

*Abbreviations*: *ED* emergency department, *N* number, *sd* standard deviation, *IQR* interquartile range, *h* hours, *BP* blood pressure, *°C* degrees celcius, *NRS* numeric rating scale

^a^A unique patient was defined as the first presentation of a patient to our ED in 2012

^b^Missing data (%): Systolic BP 768 (23.3), Heart rate 719 (21.8), Respiratory rate 1482 (45.0), Temperature 1077 (32.7), Pain score 173 (5.3), Haemoglobin 831 (25.3), Thrombocytes 1576 (47.9), Leukocytes 831 (25.3), C-reactive protein 945 (28.7), Sodium 873 (26.5), Potassium 1021 (31.0), Creatinine 873 (26.5), Urea 878 (26.7), Troponin T 1539 (46.8), Glucose 908 (27.6)

^c^Reference ranges for laboratory results: Haemoglobin male 8.5-11.0 mmol/L; female 7.5-10.0 mmol/l, Thrombocytes 150-400*10^9^/L, Leukocytes 4.00-10.00*10^9^/L, C-reactive protein 0.0-5.0 mg/L, Sodium 136-144 mmol/L, Potassium 3.6-4.8 mmol/L, Creatinine 64-104 μmol/L, Urea 2.5-7.5 mmol/L, Troponin T 0.000-0.050 μg/L, Non-fasted glucose 3.1-11.0 mmol/L

Regression analyses were performed to investigate the association between routinely assessed predictors in the ED and mortality in the first 90 days of follow-up (Table 2). A substantial portion of the univariate associations remained significant in the multivariate model i.e., age (hazard ratio [HR] 1.06, 95 % confidence interval [CI] 1.04-1.08), referral by another hospital (HR 2.74, 95 % CI 1.22-6.11), presenting complaint classified as ‘unwell’ (HR 1.99, 95 % CI 1.23-3.20), allocation to a non-surgical specialty (HR 1.55, 95 % CI 1.13-2.14), increased respiration rate (21–29 breaths per minute [bpm]: HR 1.63, 95 % CI 1.06-2.52; ≥30 bpm: HR 2.21, 95 % CI 1.25-3.92), decreased oxygen saturation (91–94 %: HR 1.63, 95 % CI 1.16-2.31; ≤90 %: HR 1.96, 95 % CI 1.19-3.23), hypothermia (HR 2.27, 95 % CI 1.28-4.01), fever (HR 0.43, 95 % CI 0.24-0.75), high pain score (HR 1.55, 95 % CI 1.03-2.32) and the indication to perform blood tests (HR 3.44, 95 % CI 2.13-5.56).

**Table 2:** Cox regression model for the association between predictors and 90-day mortality in older patients visiting the ED

		Univariate		Multivariate	
ED characteristics	Events^a^ (Total)	HR (95 % CI)	*P*-value	HR (95 % CI)	*P*-value
Age	347 (3291)	1.06 (1.04–1.08)	**<0.001**	1.06 (1.04–1.08)	**<0.001**
Sex					
Female	173 (1748)	ref	ref	ref	ref
Male	174 (1543)	1.14 (0.93–1.41)	0.219	1.15 (0.92–1.43)	0.231
Time of ED visit					
Day 08.00 h-15.59 h	165 (1677)	ref	ref	ref	ref
Evening 16.00 h-23.59 h	127 (1254)	1.03 (0.82–1.30)	0.799	0.98 (0.77–1.24)	0.857
Night 00.00 h-07.59 h	55 (360)	1.62 (1.19–2.20)	**0.002**	1.27 (0.91–1.78)	0.163
Way of arrival^b^					
Self-referral	58 (654)	ref	ref	ref	ref
Brought in by ambulance	157 (1159)	1.58 (1.17–2.13)	**0.003**	1.33 (0.95–1.84)	0.096
General practitioner	102 (1108)	1.04 (0.75–1.43)	0.833	0.91 (0.64–1.29)	0.596
LUMC internal	23 (338)	0.75 (0.46–1.22)	0.243	0.81 (0.49–1.35)	0.424
Other Hospital	7 (28)	3.04 (1.39–6.66)	**0.005**	2.74 (1.22–6.11)	**0.014**
Presentation					
Limb problems	37 (608)	ref	ref	ref	ref
Unwell	99 (598)	2.93 (2.01–4.28)	**<0.001**	1.99 (1.23–3.20)	**0.005**
Chest pain	20 (346)	0.96 (0.56–1.65)	0.882	0.54 (0.29–1.00)	0.051
Shortness of breath	56 (304)	3.22 (2.13–4.88)	**<0.001**	1.43 (0.83–2.45)	0.195
Abdominal pain	26 (214)	2.09 (1.26–3.45)	**0.004**	1.68 (0.98–2.89)	0.061
Collapsed	19 (168)	1.96 (1.13–3.42)	**0.017**	1.29 (0.68–2.44)	0.439
Falls	10 (122)	1.38 (0.69–2.77)	0.369	1.19 (0.58–2.45)	0.663
Wounds	8 (108)	1.22 (0.57–2.62)	0.610	1.47 (0.67–3.21)	0.332
Palpitations	3 (101)	0.49 (0.15–1.58)	0.229	0.36 (0.11–1.26)	0.110
Other	69 (722)	1.61 (1.08–2.40)	**0.019**	1.44 (0.93–2.23)	0.100
Consulting medical specialty					
Surgical	99 (1371)	ref	ref	ref	ref
Non-surgical	248 (1920)	1.85 (1.47–2.34)	**<0.001**	1.55 (1.13–2.14)	**0.007**
Systolic BP (mmHg)					
≤100	18 (109)	1.62 (1.00–2.61)	**0.049**	1.05 (0.64–1.72)	0.849
101–199	250 (2313)	ref	ref	ref	ref
≥200	16 (101)	1.52 (0.92–2.52)	0.104	1.15 (0.69–1.94)	0.589
Not measured	63 (768)	0.75 (0.57–0.99)	**0.044**	1.55 (0.79–3.02)	0.202
Heart rate (BPM)					
≤50	5 (55)	0.90 (0.37–2.17)	0.807	0.67 (0.27–1.68)	0.394
51–100	214 (2093)	ref	ref	ref	ref
101–110	28 (187)	1.48 (1.00–2.20)	**0.049**	1.20 (0.80–1.80)	0.375
111–129	27 (144)	1.93 (1.30–2.89)	**0.001**	1.46 (0.94–2.27)	0.090
≥130	12 (92)	1.29 (0.72–2.31)	0.392	1.41 (0.76–2.61)	0.277
Not measured	61 (720)	0.83 (0.62–1.10)	0.192	1.51 (0.72–3.14)	0.272
Respiration rate (bpm)					
≤8	1 (5)	2.65 (0.36–19.38)	0.337	2.07 (0.27–15.66)	0.481
9–14	33 (381)	ref	ref	ref	ref
15–20	95 (907)	1.21 (0.82–1.80)	0.343	1.15 (0.77–1.71)	0.507
21–29	68 (417)	1.95 (1.29–2.96)	**0.002**	1.63 (1.06–2.52)	**0.027**
≥30	31 (99)	4.16 (2.55–6.80)	**<0.001**	2.21 (1.25–3.92)	**0.007**
Not measured	119 (1482)	0.92 (0.62–1.35)	0.650	0.95 (0.61–1.47)	0.819
Oxygen saturation (%)					
≤90	22 (81)	3.08 (1.99–4.78)	**<0.001**	1.96 (1.19–3.23)	**0.008**
91–94	43 (218)	2.09 (1.51–2.90)	**<0.001**	1.63 (1.16–2.31)	**0.005**
≥95	218 (2217)	ref	ref	ref	ref
Not measured	64 (775)	0.84 (0.63–1.11)	0.212	1.22 (0.65–2.27)	0.534
Temperature (°C)					
≤34.9	14 (42)	3.43 (2.00–5.89)	**<0.001**	2.27 (1.28–4.01)	**0.005**
35.0–38.4	230 (2023)	ref	ref	ref	ref
≥38.5	14 (149)	0.82 (0.48–1.40)	0.461	0.43 (0.24–0.75)	**0.003**
Not measured	89 (1077)	0.72 (0.57–0.92)	**0.009**	1.12 (0.81–1.54)	0.498
Pain score (NRS)					
0–3	181 (1645)	ref	ref	ref	ref
4–6	110 (1136)	0.87 (0.68–1.10)	0.240	1.24 (0.95–1.61)	0.114
7–10	36 (337)	0.97 (0.68–1.38)	0.847	1.55 (1.03–2.32)	**0.034**
Not measured	20 (173)	1.06 (0.67–1.68)	0.811	0.93 (0.58–1.49)	0.754
Blood tests^c^					
None performed	29 (770)	ref	ref	ref	ref
Performed	318 (2521)	3.52 (2.41–5.15)	**<0.001**	3.44 (2.13–5.56)	**<0.001**

*Abbreviations: ED* emergency department, *HR* hazard ratio, *CI* confidence interval, *ref* reference category, *BP* blood pressure, *BPM* beats per minute, *bpm* breaths per minute, *°C* degrees Celcius, *NRS* numeric rating scale

Bold formatting has been used to mark statistical significant *P*-values

^a^‘Events’ represent the number of deaths in each category within 90 days after ED admittance

^b^Way of arrival was unknown in 4 patients (data not shown in table). No patients died in this category. Univariate Cox regression analysis showed HR 0.91 (95 % CI 0.80-1.04; *P* value 0.178). Multivariate Cox regression analysis showed HR 0.00 (95 % CI 0.00-9.37*10^102^; *P*-value 0.947)

^c^Blood tests included levels of haemoglobin, thrombocytes, leukocytes, C-reactive protein, sodium, potassium, creatinine, urea, troponin T and/or non fasted glucose

Table 3 demonstrates how abnormal versus normal laboratory results relate to mortality risk among patients who had an indication for performing blood tests. The majority of abnormal laboratory results show an increased hazard as compared to measurements within normal range. Strongest associations were a high level of troponin T (HR 3.26, 95 % CI 2.47-4.30), thrombocytes (HR 3.18, 95 % CI 2.11-4.80) and leukocytes (HR 2.50, 95 % CI 1.99-3.14). Patients for whom no laboratory tests were performed had a significantly decreased mortality risk in comparison with patients whose laboratory results were within reference range. For instance, hazard ratio for patients without a sodium measurement was 0.36 (95 % CI 0.26-0.52) as compared to patients with a sodium measurement within reference range.

**Table 3:** The association between laboratory results and 90-day mortality in older patients visiting the ED

		Univariate Cox regression analysis	
	Events^a^ (Total)	HR (95 % CI)	*P*-value
Haemoglobin			
Within reference range (male: 8.5-11.0 mmol/L, female: 7.5-10.0 mmol/L)	147 (1458)	ref	ref
Below reference range	158 (965)	1.66 (1.33–2.08)	**<0.001**
Above reference range	5 (37)	1.39 (0.57–3.38)	0.472
Not measured	37 (831)	0.43 (0.30–0.61)	**<0.001**
Thrombocytes			
Within reference range (150-400*10^9^/L)	188 (1402)	ref	ref
Below reference range	46 (242)	1.45 (1.05–2.01)	**0.023**
Above reference range	26 (71)	3.18 (2.11–4.80)	**<0.001**
Not measured	87 (1576)	0.39 (0.31–0.51)	**<0.001**
Leukocytes			
Within reference range (4.00-10.00*10^9^/L)	128 (1523)	ref	ref
Below reference range	11 (65)	2.10 (1.14–3.89)	**0.018**
Above reference range	171 (872)	2.50 (1.99–3.14)	**<0.001**
Not measured	37 (831)	0.52 (0.36–0.75)	**<0.001**
C-reactive protein			
Within reference range (0.0–5.0 mg/L)	88 (1102)	ref	ref
Above reference range	214 (1244)	2.25 (1.75–2.88)	**<0.001**
Not measured	45 (945)	0.58 (0.41–0.83)	**0.003**
Sodium			
Within reference range (136–144 mmol/L)	208 (1862)	ref	ref
Below reference range	65 (391)	1.53 (1.16–2.02)	**0.003**
Above reference range	37 (165)	2.14 (1.51–3.03)	**<0.001**
Not measured	37 (873)	0.36 (0.26–0.52)	**<0.001**
Potassium			
Within reference range (3.6–4.8 mmol/L)	200 (1804)	ref	ref
Below reference range	35 (162)	2.12 (1.48–3.03)	**<0.001**
Above reference range	58 (304)	1.78 (1.33–2.38)	**<0.001**
Not measured	54 (1021)	0.46 (0.34–0.63)	**<0.001**
Creatinine			
Within reference range (64–104 μmol/L)	127 (1258)	ref	ref
Below reference range	57 (475)	1.20 (0.88–1.64)	0.247
Above reference range	124 (685)	1.87 (1.46–2.40)	**<0.001**
Not measured	39 (873)	0.43 (0.30–0.61)	**<0.001**
Urea			
Within reference range (2.5–7.5 mmol/L)	95 (1199)	ref	ref
Below reference range	1 (1)	20.72 (2.88–148.92)	**0.003**
Above reference range	214 (1213)	2.34 (1.83–2.97)	**<0.001**
Not measured	37 (878)	0.52 (0.35–0.76)	**0.001**
Troponin T			
Within reference range (0.000–0.050 μg/L)	146 (1484)	ref	ref
Above reference value	77 (268)	3.26 (2.47–4.30)	**<0.001**
Not measured	124 (1539)	0.80 (0.63–1.02)	0.066
Non-fasted glucose			
Within reference range (3.1–11.0 mmol/L)	249 (2123)	ref	ref
Below reference range	2 (7)	2.83 (0.70–11.39)	0.143
Above reference range	55 (253)	2.04 (1.52–2.73)	**<0.001**
Not measured	41 (908)	0.37 (0.27–0.52)	**<0.001**

*Abbreviations*: *mmol* millimol, *L* liter, *mg* milligram, *HR* hazard ratio, *CI* confidence interval

Bold formatting has been used to mark statistical significant *P*-values

^a^‘Events’ represent the number of deaths in each category within 90 days after ED admittance

## Discussion

The main finding of the present study is that routinely, at entrance assessed, clinical parameters can be used to predict 90-day mortality in older persons admitted to the emergency department (ED). Independent predictors of 90-day mortality risk included: increasing age, referral by another hospital, disease presentation categorised as ‘unwell’, allocation to a non-surgical specialty, low respiration rate, low oxygen saturation, body temperature and the performance of blood tests. In addition, abnormal laboratory results, which become known at a later stage during an ED visit, are univariately associated with increased mortality risk. Patients for whom no laboratory tests were performed showed a decreased mortality risk.

Potential predictors of poor outcome in acutely presenting older adults have been studied before. Like in our study, increasing age was shown to associate with in-hospital mortality [21], as well as mortality risk 1 year after presentation [22]. Our research aimed at predictors known upon or soon after arrival of a patient at the ED in order to investigate their potential for new screening instruments. Other researchers also included predictors into their models that become available at a later stage during an ED visit, such as length of stay at the ED [21, 22]. Kennelly et al. found an association between arrival by ambulance and mortality, whereas our study did not [22]. Van Walraven et al. developed the hospital-patient one-year mortality risk (HOMR) model [23]. The HOMR model assesses 1 year mortality risk for adults ≥18 years who are acutely hospitalised, but it was not validated for ED visitors who were directly discharged without admittance to an inpatient ward. In addition, previous research shows that abnormal vital signs at triage associate with intensive care unit admission and in-hospital mortality in patients from the age of 16 [24] as well as in older patients from the age of 75 [25]. Furthermore, a high Modified Early Warning Score can be used to predict a worse in-hospital stay (e.g., mortality and hospitalisation) in older adults [7]. Our study demonstrates that respiration rate, oxygen saturation, body temperature and pain score associate with 90-day mortality independent of other risk factors. Systolic blood pressure and heart rate did not remain significantly associated with mortality in the multivariate model. However, anatomical and physiological changes that occur with ageing may limit older people to generate an adequate response to injury [26]. As a consequence, some vital signs may not be reliable in reflecting the actual condition of an older patient [25].

Managing older people in the ED can be complex because of atypical disease presentation, poly-pharmacy and multiple co-morbidities. Risk factors for adverse health outcomes include functional dependence, lack of social support and cognitive impairment [2]. Many risk factors and frailty screening tools such as the ‘Identification of Seniors at Risk’ have been evaluated in their ability to predict health outcome in older adults. Individually, they all lack sufficient prognostic accuracy to identify patients at high risk for poor outcome [27]. We found that routinely collected clinical parameters associate with mortality in older patients admitted to the ED. Although this is not unexpected, it implies that early assessed characteristics of an ED visit are not only of value with respect to short term outcomes, but may be useful when considering the period after discharge as well. Models including both disease specific parameters (for example respiration rate) and parameters reflecting functional and cognitive status may give rise to a more complete assessment of the older individual. Our findings lay ground for creating new prediction models using routinely collected parameters alongside frailty characteristics in order to adequately predict outcome in acutely presenting older patients. We are currently performing prospective studies to develop and validate such predictive models with respect to multiple negative endpoints such as mortality, admission rate, quality of life and functional status (www.apop.eu [28]). These prediction models should be able to detect patients at high risk for poor outcome and enable the development of appropriate interventions to improve acute medical care for older patients.

The present study was limited by its retrospective nature and could not provide reliable information on frailty characteristics such as multi-morbidity, poly-pharmacy and functional and cognitive impairment and these characteristics could not be studied in our model. However, it is unlikely that the investigated predictors in our study would change when collected in a prospective matter. Our study was set at a single centre tertiary referral hospital which may make our results less generalisable. Strong points of our study were the large sample size of over three thousand ED visits, the use of universal predictors that were likely to be free of bias and the fact that mortality is a very robust end point of which data were available for all patients through municipality records. Our study is unique in the fact that we investigated predictors early known during an ED visit which may be suitable for a screening instrument.

A proper screening instrument that identifies older patients at risk of poor outcome is the first step towards changing outcome. We aim that a screening instrument will enable us to set up special care trajectories in order to improve recovery after acute presentation at the ED. These tailored trajectories could include extra attention on rehabilitation, prevention of delirium and advanced care planning and are currently investigated in a prospective study concerning the acutely presenting older patient (‘APOP study’ [28]).

## Conclusions

Routinely collected parameters of older persons attending the ED can be used to predict 90-day mortality. This survey constitutes preparatory work towards creating a proper screening instrument for predicting and improving health outcome in acutely presenting older patients.

## Abbreviations

°C, degrees Celcius; APOP, acutely presenting older patient; BP, blood pressure; bpm, breaths per minute; BPM, beats per minute; CI, confidence interval; CPR, cardiopulmonary resuscitation; ED, emergency department; h, hours; HR, hazard ratio; IQR, interquartile range; L, liter; mg, milligram; mmol, millimol; N, number; NRS, numeric rating scale; sd, standard deviation; μmol, micromol
